# Identification of Salt-Sensitive and Salt-Tolerant Genes through Weighted Gene Co-Expression Networks across Multiple Datasets: A Centralization and Differential Correlation Analysis

**DOI:** 10.3390/genes15030316

**Published:** 2024-02-28

**Authors:** Pajaree Sonsungsan, Apichat Suratanee, Teerapong Buaboocha, Supachitra Chadchawan, Kitiporn Plaimas

**Affiliations:** 1 Program in Bioinformatics and Computational Biology, Graduate School, Chulalongkorn University, Bangkok 10330, Thailand; pajaree_s@hotmail.com; 2 Department of Mathematics, Faculty of Applied Science, King Mongkut’s University of Technology North Bangkok, Bangkok 10800, Thailand; apichat.s@sci.kmutnb.ac.th; 3 Center of Excellence in Molecular Crop, Department of Biochemistry, Faculty of Science, Chulalongkorn University, Bangkok 10330, Thailand; teerapong.b@chula.ac.th; 4 Center of Excellence in Environment and Plant Physiology (CEEPP), Department of Botany, Faculty of Science, Chulalongkorn University, Bangkok 10330, Thailand; supachitra.c@chula.ac.th; 5 Omics Science and Bioinformatics Center, Faculty of Science, Chulalongkorn University, Bangkok 10330, Thailand; 6 Advanced Virtual and Intelligent Computing (AVIC) Center, Department of Mathematics and Computer Science, Faculty of Science, Chulalongkorn University, Bangkok 10330, Thailand

**Keywords:** rice salt stress, salt tolerance, co-expression networks, WGCNA, salt-sensitive rice, salt-tolerant rice, network analysis

## Abstract

Salt stress is a significant challenge that severely hampers rice growth, resulting in decreased yield and productivity. Over the years, researchers have identified biomarkers associated with salt stress to enhance rice tolerance. However, the understanding of the mechanism underlying salt tolerance in rice remains incomplete due to the involvement of multiple genes. Given the vast amount of genomics and transcriptomics data available today, it is crucial to integrate diverse datasets to identify key genes that play essential roles during salt stress in rice. In this study, we propose an integration of multiple datasets to identify potential key transcription factors. This involves utilizing network analysis based on weighted co-expression networks, focusing on gene-centric measurement and differential co-expression relationships among genes. Consequently, our analysis reveals 86 genes located in markers from previous meta-QTL analysis. Moreover, six transcription factors, namely *LOC_Os03g45410* (*OsTBP2*), *LOC_Os07g42400* (*OsGATA23*), *LOC_Os01g13030* (*OsIAA3*), *LOC_Os05g34050* (*OsbZIP39*), *LOC_Os09g29930* (*OsBIM1*), and *LOC_Os10g10990* (*transcription initiation factor IIF*), exhibited significantly altered co-expression relationships between salt-sensitive and salt-tolerant rice networks. These identified genes hold potential as crucial references for further investigation into the functions of salt stress response in rice plants and could be utilized in the development of salt-resistant rice cultivars. Overall, our findings shed light on the complex genetic regulation underlying salt tolerance in rice and contribute to the broader understanding of rice’s response to salt stress.

## 1. Introduction

Rice (*Oryza sativa* L.) is the primary food crop in the world. Several rice cultivars, including Indica and Japonica, are consumed increasingly yearly, especially in Asian countries. In Thailand, rice is mainly planted in the northeast, which faces challenges due to salt stress. The presence of salt stress limits plant growth, reduces yield, and poses threats to food security in the country. Moreover, salinity remains a major issue in rice cultivation worldwide, as rice is particularly vulnerable to salt stress during the seedling and reproductive stages, directly impacting its growth [1,2,3,4,5]. Technological advancements and computational approaches have become widely used to enhance rice’s ability to tolerate salt stress by identifying genomic loci and specific genes associated with salt tolerance. These markers can be rapidly targeted for breeding strategies, facilitating the development of salt-tolerant rice varieties [6,7].

In the past decade, there has been extensive research on molecular markers associated with salt tolerance, which have been employed in breeding programs to increase the number of salt-tolerant rice cultivars [6,7]. Various approaches have identified several genomic markers: (1) Quantitative trait loci (QTLs) have been discovered by studying mapping populations derived from crosses between salt-sensitive and salt-tolerant varieties. For instance, in 2009, Ammar et al. identified 25 QTLs in a mapping population of F2 plants and their corresponding F3 families resulting from crossing CSR27 (salt-tolerant) and MI48 (salt-sensitive) at the seedling, vegetative, and reproductive stages [8]. Additionally, in 2010, Thomson et al. identified 17 QTLs by crossing ‘Pokkali’ (salt-tolerant) with IR29 (salt-sensitive) at the seedling stage [9]. (2) Genome-wide association studies (GWAS) have been employed to identify variations in loci associated with specific traits, including allelic variations in candidate genes. GWAS is a powerful approach for detecting multiple alleles at the same site and is used to identify several loci and novel candidate genes associated with salt tolerance at various stages. For example, Lekklar et al. identified 146 candidate genes on all chromosomes through GWAS mapping associated with salt tolerance in 104 Thai rice varieties at the flowering stage. Among the candidate genes, 27% are novel [10]. In 2020, Yuan et al. found 21 QTLs associated with salt tolerance in 664 indica and japonica rice varieties. Two candidate genes, *OsSTL1* and *OsSTL2*, were confirmed to be associated with salt stress using sequence analysis [11]. In 2021, Nayyeripasand et al. used GWAS to identify known and novel candidate genes associated with salt stress on ten chromosomes, based on a study of 155 rice cultivars at the vegetative stage [12]. (3) Genes related to salt tolerance in rice have been identified by comparing omics data, such as transcriptome, proteome, and metabolome, using high-throughput techniques. These approaches have proven to be powerful tools for discovering numerous potential genes associated with salt tolerance. For example, in 2021, Sonsungsan et al. constructed two-state networks under control and salinity conditions using transcriptomes of Thai rice at various time points after salt-stress treatment. They uncovered numerous known and novel candidate genes that may serve as hub genes by comparing networks between control and salt stress conditions [13]. Similarly, in the same year, Cartagena et al. compared transcriptome profiles of three root types (L-type lateral roots, nodal roots, and S-type lateral roots) in ‘Mulai’ (salt tolerant) and IR29 (sensitive rice) rice in response to salinity stress. They identified differentially expressed genes specific to salinity stress for each root type [14].

Indeed, despite identifying numerous genomic markers, such as loci and candidate genes associated with salt tolerance in various rice cultivars, our understanding of the complete mechanism underlying salt tolerance in rice remains limited [7]. The involvement of multiple genes in this complex process necessitates further research to comprehensively capture the salt-tolerant genes present in different rice varieties. Continued exploration and study of these genes are essential for making significant strides in improving rice’s salt tolerance, addressing the challenges of salt stress, and enhancing rice productivity.

To speed up the discovery of candidate genes, network analysis has emerged as a powerful and complementary approach. It aids in understanding how genes interact to respond to salt stress and identifies crucial genes that potentially exert control over other genes within the system [15,16,17]. Here, we aimed to identify key genes among differentially expressed genes for salt-sensitive rice and salt-tolerant rice cultivars under control and salinity conditions. Gene expression data from microarray experiments and high-throughput RNA sequencing were collected for both salt-sensitive rice and salt-tolerant rice cultivars. After that, we first compared the gene expression profiles of these two contrasting cultivars under the same conditions and constructed weighted gene co-expression networks. The weighted gene co-expression network analysis was then applied to identify high-centrality genes in both networks. Moreover, we analyzed genes involved in significant changes in correlations between gene pairs to qualify key genes as transcription factors. Finally, we conducted gene ontology and functional analysis for the identified genes in each salt-sensitive and salt-tolerant rice cultivar. The results were compared to better understand the molecular mechanism underlying those two cultivars.

## 2. Results

**Figure 1:**
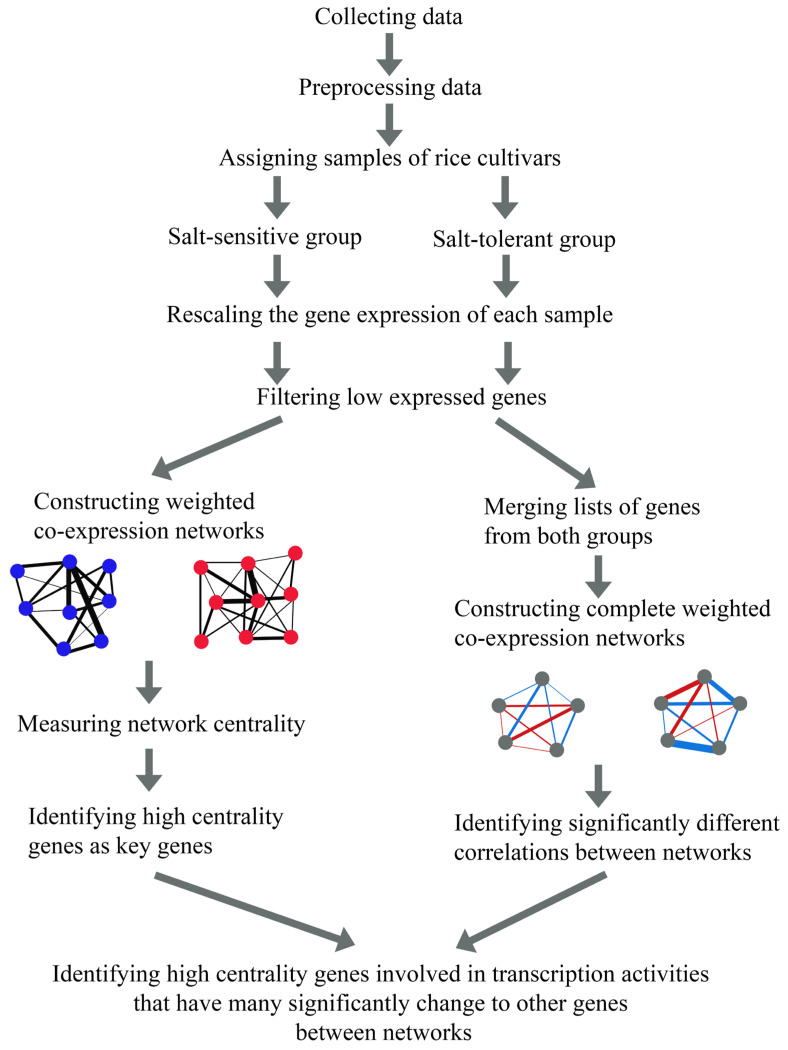
Flowchart showing different steps of the analysis pipeline used to identify the candidate genes involved in salinity tolerance in rice. The gene expression data from microarray and RNA sequencing techniques of the salt-sensitive and salt-tolerant rice cultivars were integrated to construct weighted gene co-expression networks for comparing node centrality and significant differences in correlation between networks. Blue nodes represent highly expressed genes of the salt-sensitive rice and red nodes represent highly expressed genes of the salt-tolerant rice. Blue and red edges represent positive and negative correlations, respectively. Gene involved in transcription activities that have high-centrality and exhibit significant change in correlations were captured.

In this study, we compared the weighted co-expression networks of salt-sensitive and salt-tolerant rice cultivars under both control and salinity conditions and identified key differentially expressed genes between the two networks. Figure 1 illustrates our analysis pipeline, which begins by collecting gene expression datasets from both micro-array and RNA sequencing experiments related to salinity tolerance in rice from the NCBI’s Gene Expression Omnibus (GEO database; https://www.ncbi.nlm.nih.gov/geo/) (accessed on 28 August 2023). Next, data preprocessing was performed on each dataset, including aligning the probe IDs of microarray datasets with gene names in the MSU system, which is the Rice Genome Annotation Project funded by National Science Foundation (NSF) in the USA. Initially located at The Institute for Genomic Research at Michigan State University (MSU), it is now housed at the University of Georgia (UGA) (http://rice.uga.edu/) (accessed on 14 October 2023), calculating the average for genes from multiple probes, and combining microarray and RNA-seq data. Then, each sample of the rice cultivars was assigned to either the salt-sensitive or salt-tolerant group. To filter out low-expressed genes, we rescaled the gene expression of each sample from 0 to 100 and selected genes with an expression value over 1 in at least 50% of samples for each group. Next, we constructed and analyzed weighted co-expression networks for salt-sensitive and salt-tolerant rice cultivars using two approaches. The first involved a gene co-expression network for each rice group across all conditions, using weighted correlation network analysis (WGCNA) [18] and identifying key differentially expressed genes between networks based on network centrality measurements. The second task involved constructing a gene co-expression network across all conditions for genes found in both groups and identifying key genes based on significantly different correlations between networks using Diffcorr package version 0.4.3 in R [19]. Finally, we investigated significant changes in correlations to other genes between the two networks of high-centrality genes involved in transcription activities. These genes may play an important role in salinity responses in rice by regulating other genes in the mechanism.

### 2.1. Characteristics of the Weighted Gene Co-Expression Networks

**Figure 2:**
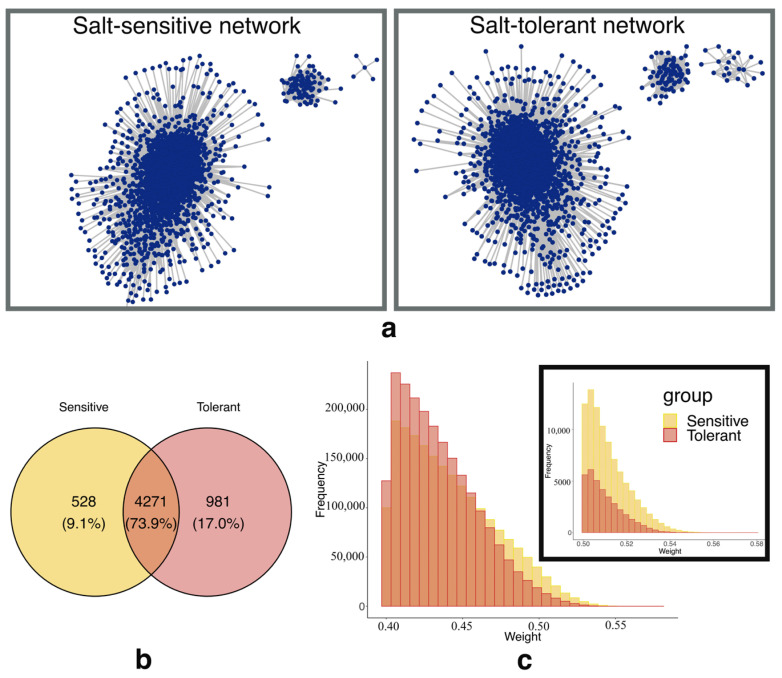
Network analysis based on WGCNA. (**a**) Gene co-expression network of the salt-sensitive and salt-tolerant rice groups. (**b**) Venn diagram indicates the number of genes in each network. (**c**) Histogram comparing the edge weights between salt-sensitive and salt-tolerant networks.

Based on the WGCNA method [18,20], the salt-sensitive network consisted of 4799 genes and 2,028,808 edges. The minimum and maximum weights on the edges were 0.4 and 0.5784985, respectively. In comparison, the salt-tolerant network consisted of 5252 genes and 2,144,118 edges. The minimum and the maximum weight on the edges were 0.4 and 0.5512337, respectively. Both networks contain three sub-networks, but the salt-tolerant network has more nodes and edges. These findings indicated that the salt-tolerant network is more complex than the salt-sensitive network, as shown in Figure 2a. The networks shared 4271 genes in common (Figure 2b). The salt-sensitive network exhibited higher edges with weights exceeding 0.5 compared to the salt-tolerant network (Figure 2c). These observations suggest that genes within the salt-sensitive network display more similar expression patterns than those within the salt-tolerant network, despite the salt-tolerant network containing a higher number of genes than the salt-sensitive network.

### 2.2. Network Parameters and Centralities

The network data of the salt-sensitive and the salt-tolerant groups were computed with a comprehensive set of topological parameters and centralities using Cytoscape version 3.10.1 [21]. Network parameters are shown in Table 1. The average number of neighbors per node in the salt-sensitive network is greater than in the salt-tolerant network. This indicates that many genes in the salt-sensitive network show more similar expression patterns (are connected) than genes in the salt-tolerant network. Moreover, the salt-sensitive network has a slightly greater number of network diameters and network radius. Additionally, the maximum of all diameters of its connected components and the network density of the salt-sensitive network are larger than in the salt-tolerant network. These findings indicate that the salt-sensitive network is characterized by greater density and stronger connectivity than the salt-tolerant network. This implies that genes in the salt-tolerant rice under salt-stress conditions may be activated or inhibited by some genes in mechanisms, leading to changes in expression patterns and the loss of connection to other genes. Furthermore, genes within the salt-tolerant network demonstrate a higher tendency to cluster together and function effectively under given conditions (the network clustering coefficient), compared to genes within the salt-sensitive network. Additionally, the salt-tolerant network displays a slightly higher level of network heterogeneity, indicated by the presence of hub nodes, than the salt-sensitive network.

Rather than considering the basic network properties as shown in Table 1, the analysis focused on node centrality to detect important nodes for the overall network structure. This aimed to clarify which centrality measures would be suitable techniques for distinguishing changes in connections of a certain gene under normal conditions compared to salinity conditions. We calculated four centralities (degree, betweenness, closeness, and clustering coefficient) for each gene in each network. In principle, these four centralities detect different aspects of a node within the network.

**Figure 3:**
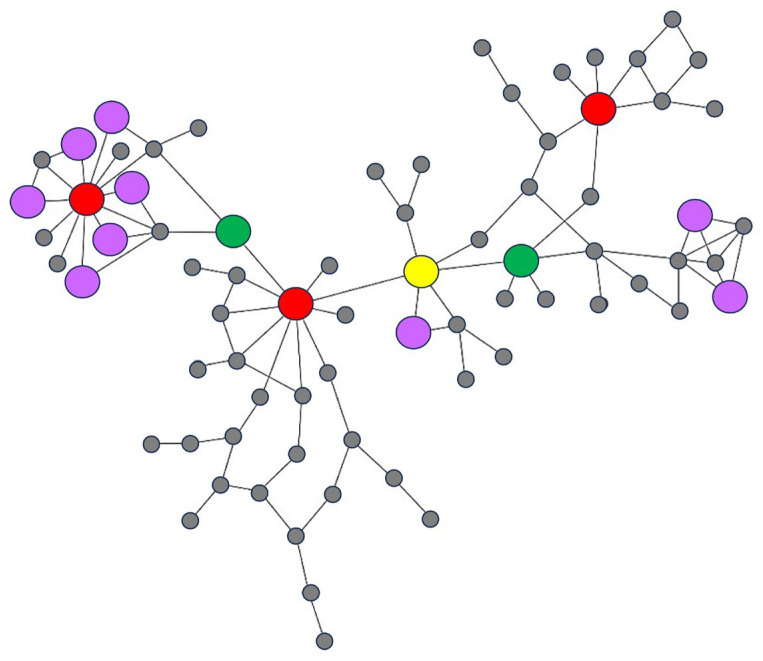
An example of high centrality nodes in a subnetwork. The red, yellow, green, and purple nodes indicate genes with high degree, high betweenness, high closeness, and high clustering coefficient, respectively.

To better understand each centrality in the network, we selected a subnetwork of the salt-tolerant network that contained genes with degrees 1 to 20 and edges with weight in the 0.5–0.52 intervals, as shown in Figure 3. The four centralities of each gene were measured with Cytoscape (version 3.10.1) software. In the subnetwork, red nodes represent genes that are connected to many other genes in the network, making them central as they have many neighbors. The yellow node exhibits a high betweenness value, acting as a ‘bridge’ to facilitate the flow of information among genes in the network. Deleting this gene from the network would disconnect it and separate it into two components. Green nodes are well-connected and in close proximity to other genes, allowing them to quickly spread information to other genes. Purple nodes possess high clustering coefficients, enabling them to form densely connected clusters within the network; this means that they are connected to many other nodes and their neighbors are also interconnected.

**Figure 4:**
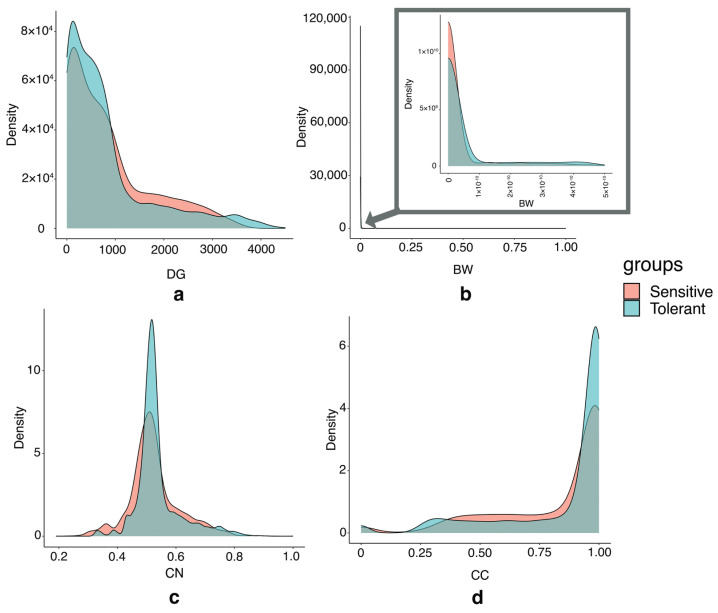
The distribution plot of centralities compared between salt-sensitive and salt-tolerant networks. The *x*-axis represents the value of (**a**) degree centrality (DG), (**b**) betweenness centrality (BW), (**c**) closeness centrality (CN), and (**d**) clustering coefficient (CC). The *y*-axis represents the density of the distribution.

We then compared the centrality distributions between the salt-sensitive network and the salt-tolerant network. Similar patterns were observed when comparing the distribution of centralities in both networks. In the salt-sensitive network, the node degrees ranged from 1 to 3674. Genes with the highest degrees were adjacent to approximately 76.56% of the network. Most nodes exhibited relatively moderate degrees, with a median of 589 and a mean of 845.51. In contrast, the salt-tolerant network had node degrees ranging from 1 to 4505. Genes with the highest degrees were adjacent to approximately 85.76% of the network. Similarly, most nodes had relatively moderate degrees, with a median of 533 and a mean of 816.50. The degree centrality distributions in both networks were right skewed with long tails (Figure 4a). This indicates that most genes had relatively small degrees, while a few nodes had extremely high degrees.

The distribution of betweenness centrality in both networks was highly dense and close to zero (Figure 4b). In the salt-sensitive network, the first quartile, mean, median, and maximum are 0, 0.0005239, 0.0000010, and 1, respectively. Similarly, in the salt-tolerant network, the first quartile, mean, median, and maximum is 0, 0.0005283, 0.0000005, and 0.3668305, respectively. Only one gene (*LOC_Os04g52450* encoding γ-Aminobutyric acid transaminase, BW = 1) in the salt-sensitive network had a betweenness value greater than 0.5, while no genes in the salt-tolerant network exceeded this threshold. This suggests that the betweenness may not be a reliable criterion for identifying critical genes in this study.

Regarding closeness centrality, the distribution was similar between the two networks (Figure 4c). A few genes in both networks were found to be closely connected to all other nodes. In the salt-sensitive network, only one gene (*LOC_Os04g52450*) had an average shortest path to all nodes of 1, while no gene exhibited this characteristic in the salt-tolerant network. However, more genes in the salt-tolerant network had high closeness centrality, greater than or equal to 0.8. We found that 17 and 50 genes had high closeness centrality in the salt-sensitive and salt-tolerant networks, respectively. This suggests that genes in the salt-tolerant network can spread information more efficiently than in the salt-sensitive network.

The clustering coefficient distribution in both networks was left-skewed, with a maximum value of 1 in both cases (Figure 4d). The median and the third quartile were 0.9651 and 0.9966 in the salt-sensitive network and 0.9712 and 0.9975 in the salt-tolerant network. Furthermore, it was found that 672 genes in the salt-sensitive network and 785 genes in the salt-tolerant network formed sub-complete graphs, indicated by a clustering coefficient of 1. These genes exhibited small-world properties, suggesting that many genes in the network tend to cluster together and may have similar functions in certain pathways in the salt-sensitive and salt-tolerant rice varieties.

### 2.3. Genes with Highly Different Centrality Scores in Salt-Sensitive versus Salt-Tolerant Networks

Based on the distribution of centrality results, we found that betweenness centrality and clustering coefficient may not serve as suitable criteria for comparing essential genes between networks. Both networks exhibited significantly low betweenness centrality for their respective genes, and notably high clustering coefficient values for many genes. As a result, we directed our focus to compare genes with high degrees or high closeness of genes between the networks.

#### 2.3.1. Degree Centrality

The degree centrality (DG) of the weighted gene co-expression network in this study indicates the number of co-expressions a gene possesses. Higher values indicate that the gene exhibits greater similarity in expression patterns with other genes across all the samples in the networks. The distribution of degree centrality (Figure 4a) of both networks shows a few nodes with high degrees. Therefore, we focused on examining how high-degree genes in the salt-tolerant network differ from the salt-sensitive. In this study, we focused on genes connected to at least 50% of other genes in the network as high-degree genes. The lists of high-degree genes, their degree centrality, and a description of each gene of both networks are shown in Table S1.

We found 401 high-degree genes in the salt-sensitive network (2400 < DG < 3674) (Table S1.1). These genes indicated a strong connection to other genes in the network. Among these genes, only one gene was not a node in the salt-tolerant network (DG = 0), and 24 genes showed weak connections, connecting to at most 10% of other genes in the salt-tolerant network (0 < DG < 525). In comparison, we found 389 high-degree genes in the salt-tolerant network (2633 < DG < 4505) (Table S1.2). These genes indicated a strong connection to other genes in the network. Among these genes, 25 were not in the salt-sensitive network (DG = 0), and 29 showed weak connections, connecting to at most 10% of other genes in the salt-sensitive network (0 < DG < 441). A list of high-degree genes that are present only in the sensitive or salt-tolerant network is shown in Table 2. This finding suggests that these genes play distinct roles between salt-sensitive and salt-tolerant rice cultivars. Investigating why or how these genes are connected to numerous genes in one network but not in the other would be highly interesting. The regulation of these genes may exhibit high specificity in rice cultivars’ response to salt stress.

Moreover, both networks shared 198 high-degree genes, indicating that these genes serve as central components in both networks. These genes likely possess the capability to respond to salt stress in both salt-sensitive and salt-tolerant rice cultivars.

#### 2.3.2. GO Analysis of High-Degree Genes

We performed GO analysis on high-degree genes in each network. From the GO analysis of the high-degree genes of the salt-sensitive network, we found that only 283 genes were annotated from the MSU (version 7.0) database. These genes are significantly enriched in 68 GO terms (Table S2.1). In contrast, only 255 genes were annotated from the MSU (version 7.0) database in the salt-tolerant network. Despite the fewer annotated high-degree genes in the salt-tolerant network compared to the salt-sensitive network, they were significantly enriched in more GO annotation, totaling 88 GO terms (Table S2.2). We found that 60 GO terms were commonly significant among genes in both networks. Interestingly, we observed that high-degree genes in both networks were involved in transport and translation activities, with slight differences in the number of high-degree genes between networks. These activities include transport (GO:0006810), intracellular transport (GO:0046907), protein transport (GO:0015031), intracellular protein transport (GO:0006886), vesicle-mediated transport (GO:0016192), translation (GO:0006412), translation initiation factor activity (GO:0003743), and translation factor activity, nucleic acid binding (GO:0008135).

The high-degree genes in the salt-tolerant network exhibit significant enrichment in 29 GO annotations that differ from the significant GO annotations of high-degree genes in the salt-sensitive network. Many of these GO annotations are associated with various activities, such as nucleotide binding (GO:0000166), biological regulation (GO:0065007), signal transmission (GO:0023060), signal transduction (GO:0007165), tRNA aminoacylation (GO:0043039), aminoacyl-tRNA ligase activity (GO:0004812), ligase activity (GO:0016875 and GO:0016876), translational initiation (GO:0006413), transcription from RNA polymerase II promoter (GO:0006366), RNA polymerase II transcription factor activity (GO:0003702), and nuclear transport (GO:0051169). In comparison, only nine of the significant GO annotations from the high-degree genes in the salt-sensitive network differ from those in the high-degree genes in the salt-tolerant network, such as catabolic process (GO:0009056), endoplasmic reticulum (GO:0005783), proteasome core complex (GO:0005839), threonine-type endopeptidase activity (GO:0004298), and threonine-type peptidase activity (GO:0070003).

#### 2.3.3. Closeness Centrality

From the distribution of centralities, the closeness centrality (CN) reveals a small number of genes with high closeness centrality in both the salt-tolerant and salt-sensitive networks (Figure 4c). Closeness centrality measures the distance between two genes in the network. Genes with high closeness demonstrate the ability to have the shortest paths to other genes in the network. They may play an essential role as root nodes in the network, facilitating the spread of information and communication with other genes in the mechanism. This study focused on genes with high closeness centrality (CN ≥ 0.75) as high-closeness genes in the networks. The lists of genes, the description of each gene with high closeness centrality, and the description of each gene of both networks are shown in Table S3.

In the salt-sensitive network, 81 genes exhibited a high closeness centrality value (Table S3.1). Only four genes showed low closeness values (CN ≤ 0.5) in the salt-tolerant network. In comparison, 153 genes had a high closeness centrality value in the salt-tolerant network (Table S3.2). Only seven genes showed a low closeness value (CN ≤ 0.5) in the salt-sensitive network. A list of high-closeness genes indicated in either the sensitive or salt-tolerant network is shown in Table 3. Moreover, 41 genes exhibited a high closeness centrality value in both networks. These findings indicated that genes playing a role in closely or quickly communicating with other genes differ between salt-sensitive and salt-tolerant rice cultivars. Many genes have lost the ability to spread information to other genes compared to the two contrasting rice cultivars.

#### 2.3.4. GO Analysis of High-Closeness Genes

We performed GO analysis on high-closeness genes for each network. From the GO analysis of high-closeness genes of the salt-sensitive network, we found only 64 genes were annotated from the MSU (version 7.0) database. These genes are significantly enriched in 24 GO terms (Table S4.1). In contrast, we found 109 genes were annotated in the MSU (version 7.0) database in the salt-tolerant network. These genes are significantly enriched in 49 GO terms (Table S4.2). We found that 13 GO terms were commonly significant among genes in both networks. Additionally, high-closeness genes in both networks were involved in many catabolic processes, such as cellular macromolecule catabolic process (GO:0044265), macromolecule catabolic process (GO:0009057), cellular catabolic process (GO:0044248), and catabolic process (GO:0009056).

The high-closeness genes in the salt-tolerant network exhibit significant enrichment in 36 GO annotations that differ from the significant GO annotations of high-closeness genes in the salt-sensitive network. These GO terms are involved in transport activities, signal transduction, signaling processes, translation, GTP activities, and phosphatase activities, such as intracellular transport (GO:0046907), protein transport (GO:0015031), transport (GO:0006810), vesicle-mediated transport (GO:0016192), signal transduction (GO:0007165), small GTPase mediated signal transduction (GO:0007264), signal transmission (GO:0023060), signaling process (GO:0023046), translation (GO:0006412), regulation of biological process (GO:0050789), GTP binding (GO:0005525), small GTPase mediated signal transduction (GO:0007264), guanyl nucleotide binding (GO:0019001), hydrolase activity, acting on acid anhydrides (GO:0016817), nucleoside-triphosphatase activity (GO:0017111), and pyrophosphatase activity (GO:0016462). Whereas high-closeness genes in the salt-sensitive network were significantly enriched in only 11 GO annotations that differ from high-closeness genes in the salt-tolerant network, such as photosynthesis, light harvesting (GO:0009765), photosynthesis (GO:0015979), carboxylic acid metabolic process (GO:0019752), oxoacid metabolic process (GO:0043436), cellular ketone metabolic process (GO:0042180), organic acid metabolic process (GO:0006082), and cofactor binding (GO:0048037).

### 2.4. Identification of High-Centrality Genes Associated Meta-QTLs in Rice

To identify genes potentially involved in salinity tolerance in rice based on previous QTL studies, we focused on the identification of high-centrality genes within genome regions through QTL meta-analysis. This approach aimed to detect candidate genes frequently implicated in responding to salinity stress across various studies. SSR markers corresponding to the meta-QTL were retrieved from [22]. Researchers in the study collected all reported QTLs related to salinity tolerance in rice and identified the meta-QTL with a 95% confidence interval (CI) using the lowest Akaike information criterion (AIC) values. A total of 46 meta-QTL intervals were identified in their study.

**Figure 5:**
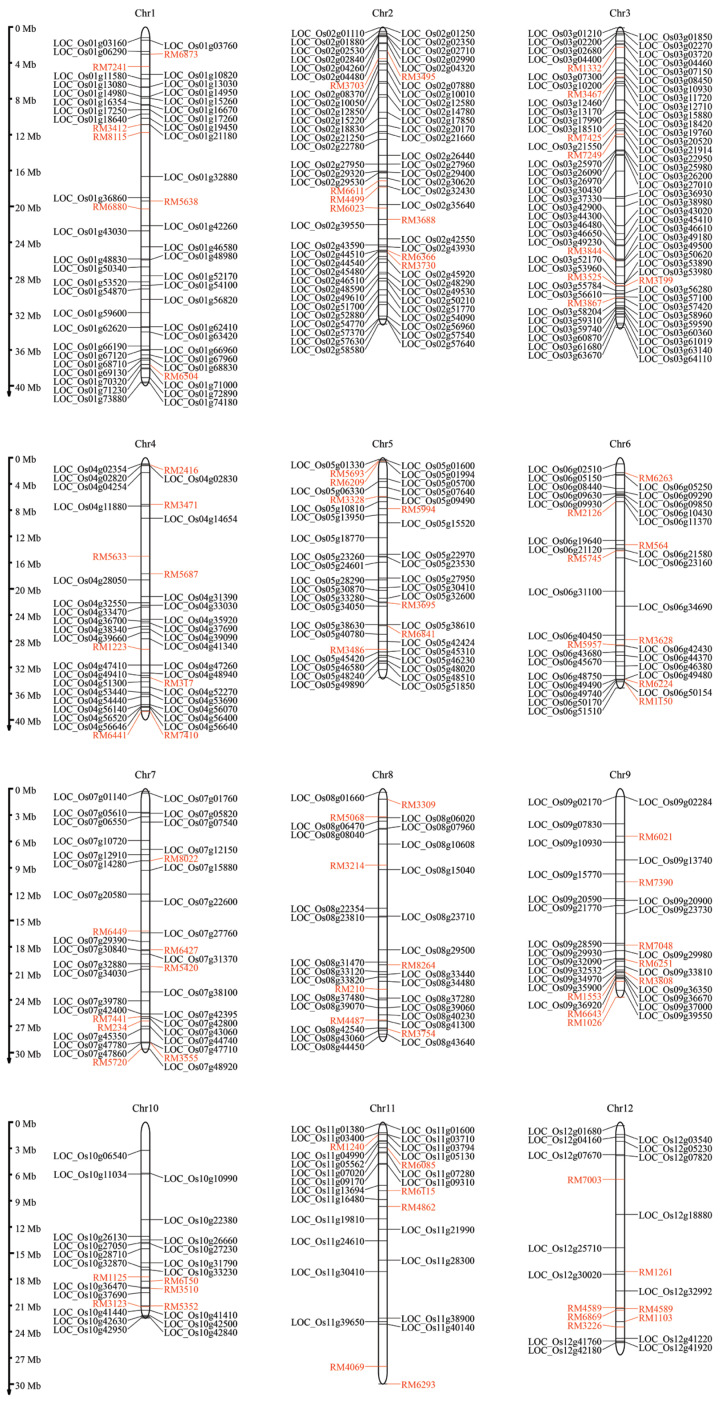
The location of each high-centrality gene from the salt-tolerant network on every chromosome is illustrated. Vertical lines on the left indicate the size of each chromosome in Mb on the consensus map, with marker names shown in red. Genes located within the meta-QTL positions associated with rice salt tolerance [22] are indicated in black.

Figure 5 shows the mapping of our high-centrality genes onto meta-QTLs to preliminarily determine candidate genes. The meta-QTL markers were distributed on all chromosomes, with 86 high-centrality genes located within the marker intervals of the meta-QTLs (refer to Table S5). These genes represent promising potential candidates whose functions can be validated to enhance salt tolerance in rice. Furthermore, many high-centrality genes were not situated within the meta-QTL intervals but were documented in previous literatures. This information can serve as a guide for further analysis of these predicted genes.

### 2.5. Identification of Candidated Genes in Thai Rice

In Thai rice, the salt-tolerant rice chromosome segment substitution lines (CSSLs) of ‘Khao Dawk Mali 105’ (‘KDML105’) previously identified salt tolerance genes in the putative drought tolerance genetic region on chromosome 1 [23,24]. Some genes between RM1003 and RM3362 markers were investigated in CSSL16. The expression levels *LOC_Os01g59080* and *LOC_Os01g64960* were significantly up-regulated induced by salt stress in CSSL16 [23]. Moreover, some genes located between RM212 and RM3362, including *LOC_Os01g61010* (*OsNodulin*), *LOC_Os01g64870*, *LOC_Os01g66890* (*OsBTBZ1*), *LOC_01g67370*, *LOC_Os01g71190*, *LOC_Os01g72210* (*OsERD*), and *LOC_01g73110*, had their expression levels investigated under salt-stress conditions in CSSLs lines [24]. From the previous study, only few genes were investigated their role in abiotic stress responses in Thai rice.

**Figure 6:**
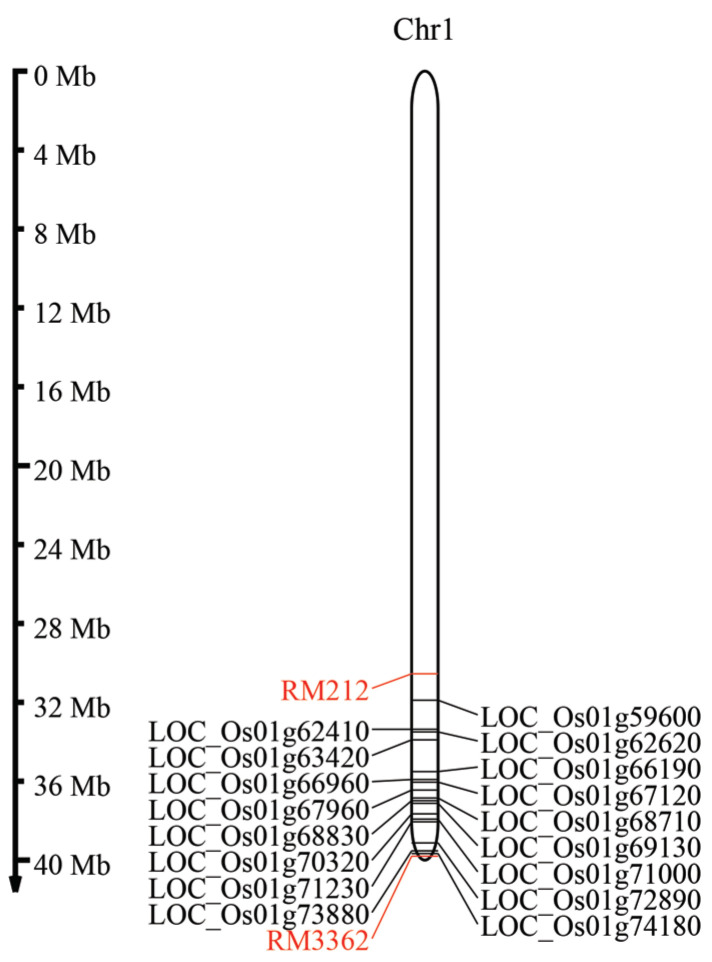
The location of high-centrality genes from the salt-tolerant network on chromosome 1 within the RM212-RM3362 interval is depicted. Vertical lines on the left represent the size of the chromosome in Mb on the consensus map, with marker names shown in red. Genes are indicated in black.

For the development of salt-tolerant Thai rice, we identified high-centrality genes in the RM212-RM3362 region on chromosome 1. We found that 17 genes are located within the marker intervals, as shown in Figure 6. Among them, three genes were reported to be involved in stress responses in rice. *LOC_Os01g62410* (*OsMYB3R-2*) was found to exhibit enhanced stresses in *Arabidopsis* and rice [25,26]. *LOC_Os01g63420* (*OsCOI1*) was found to play an important role in rice defense responses to drought tolerance [27]. *LOC_Os01g72890* (*OsSR45*) was found to interact with *OsFKBP20-1b* to play an essential role in post-transcriptional regulation of abiotic stress response [28]. The remaining genes require analysis to investigate their function in rice salt-stress response.

### 2.6. Identification of Transcription Factors Involving Various Significantly Different Correlations between the Networks

Rice responds to salt-stress conditions by regulating gene expression, which leads to up-regulation or down-regulation of numerous proteins and associated mechanisms. These regulatory processes are mediated by transcription factors (TFs), including *MYB*, *WRKY*, *NAC*, *bHIH*, etc. [29,30,31,32,33,34,35]. Therefore, our focus was on genes associated with transcription activities, drawn from both the high-degree and high-closeness genes of both networks. This approach allows us to examine how these genes interact with other genes and how they differ between salt-sensitive and salt-tolerant rice cultivars.

**Figure 7:**
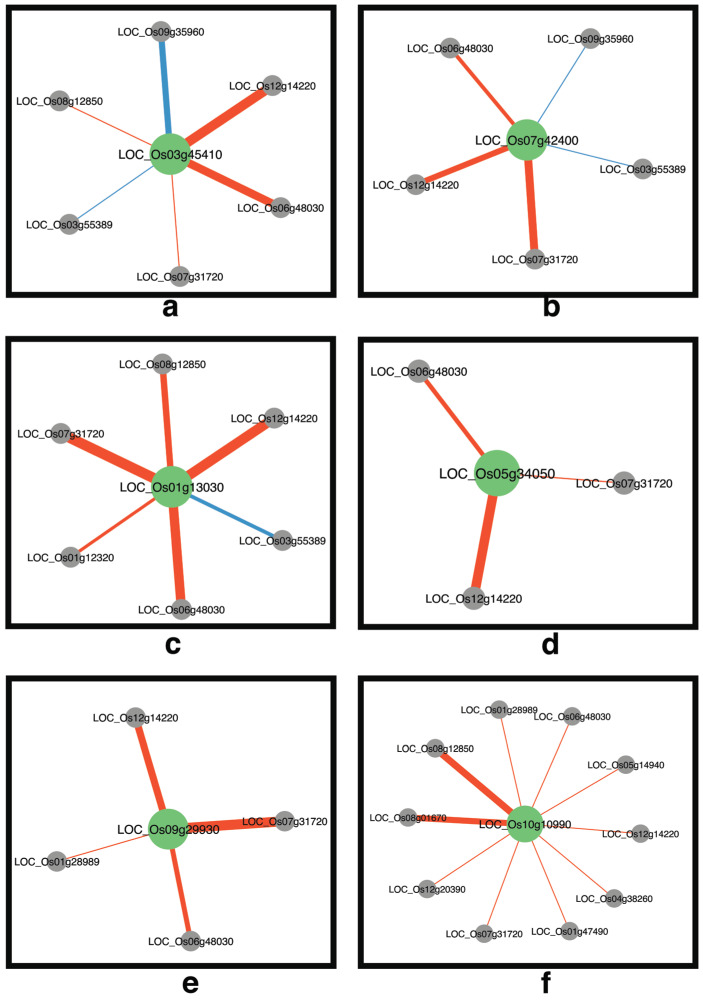
Transcription factors exhibiting high centrality in the salt-tolerant network. Each network shows the significantly different changes in correlations of (**a**) *LOC_Os03g45410*, (**b**) *LOC_Os07g42400*, (**c**) *LOC_Os01g13030*, (**d**) *LOC_Os05g34050*, (**e**) *LOC_Os09g29930*, and (**f**) *LOC_Os10g10990* with other genes across networks. Each node represents a gene, with green nodes indicating transcription factors and grey nodes indicating other genes within the network. Edge denotes significantly different correlations of at least 0.7 between networks, with red indicating negative different correlations and blue indicating positive different correlations. The weight of the edges represents the range of correlation differences, from low (thin edges) to high (thick edges).

We focus on genes in the list that are involved in transcription activities, including transcription (GO:0006350), transcription regulator activity (GO:0030528), transcription, DNA-dependent (GO:0006351), and transcription from RNA polymerase II promoter (GO:0006366). We identified ten genes involved in transcription activities in the salt-sensitive network and 20 genes in the salt-tolerant networks. Notably, we found that five genes were common to both networks. To investigate the change in gene expression levels across conditions of genes involved in transcription activities to other genes between salt-sensitive and salt-tolerant rice cultivars, we considered significantly different correlations between the two networks of all those genes that changed the correlation of at least 0.7 to avoid the difference between two low correlations, e.g., cor (sensitive) = 0 and cor (tolerant) = 0.6. From the list of 20 genes involved in transcription activities, we found only 13 genes exhibited significantly different correlations between the two networks (Table S6). We found six transcription factors with high centrality in the salt-tolerant network. Two of these genes are common in the salt-sensitive network. The significantly different correlations between networks of those genes are shown in Figure 7. Negative different correlations indicate that the correlation coefficient in the salt-sensitive is lower than the correlation coefficient in the salt-tolerant network. In contrast, positive different correlations indicate that the correlation coefficient in the salt-sensitive is higher than the correlation coefficient in the salt-tolerant network.

*LOC_Os03g45410* (*OsTBP2*) is a TATA-binding protein (TBP). We found that *OsTBP2* significantly alters the expression profile between genes and six other genes in the networks; two genes have a positive different correlation, and four genes have a negative different correlation (Figure 7a). *OsTBP2* has not yet been studied in response to salt stress. However, genes in the TBP family were reported to interact with the transcription factor IIB (TFIIB) as key components of the RNA polymerase II transcription initiation complex [36,37]. Many studies indicate that TBP genes are associated with plant stress responses [38,39,40,41]. In rice, *OsTBP2.1*, a ubiquitous transcription factor, was reported to be related to positively regulating growth and grain yield and influencing gene expression regulating the transcription of *OsNRT2.3a* and *OsNRT2.3b*, which affects nitrogen uptake in rice [42]. Moreover, *OsTBP2.2* was reported to be involved in the regulation of the photosynthesis processes in the rice response to drought conditions tested by knockdown mutant [43]. The transcriptome analysis of the *OsTBP2.2* mutant revealed the effect on *OsRCCR* gene expression, which was predicted as one of the key genes with a role in salt tolerance by the weighted gene co-expression network of the salt tolerance rice cultivar, ‘Luang Pratahn’ rice [13]. *OsTBP2* could interact with *RF2a*, a rice bZIP transcription factor, and showed synergistic effects on in vitro transcription activity of the Rice tungro bacilliform virus (RTBV) promoter [44].

Based on the network with *OsTBP2* connection, a strong connection is predicted with the downregulation of *LOC_Os09g35960*, encoding RabGAP/TBC domain containing protein. *OsTBP2* is also positively connected with *LOC_Os07g31720*, encoding *OsGAP*, which is orthologous to centaurin and annotated as a C2-domain abscisic acid-related protein. Both centaurin and RabGAP/TBC domain-containing proteins were reported to interact with PtdIns(3,4,5)P3 [45]. Small GTPase has been reported to be involved in responses to both biotic and abiotic stress (for review; [46]). The Ypt/Rab family of small G-proteins is important in regulating vesicular transport [47]. RAB GTPases are important proteins that influence various aspects of membrane traffic and play a role in abiotic stress response [48]. This is consistent with our predicted connection between *OsTBP2* and *LOC_Os08g12850* (Figure 7a), encoding reticulon-like protein, which is a protein associated with the endoplasmic reticulum (ER). During salt stress, ER-associated degradation (ERAD) via the ubiquitin/proteasome process was reported to degrade misfolded and unfolded proteins in the ER. Mutations in *Arabidopsis* ERAD genes, Sel1l/hrd3, hrd1a/hrd1b, and Os9, caused less tolerance to salt stress [49,50]. Moreover, the prediction in this module reveals the interaction between *OsTBP2* and *LOC_Os03g55389*, encoding casein kinase II (CK2) alpha subunit, which is involved in heading date and photoperiod sensitivity (RAB-DB annotation). This suggests that *OsTBP2* may be involved in the response at the reproductive stage via CK2 signaling pathway. CK2 proteins are involved in many biological processes, including abiotic stress response. Downstream genes were reported (for review; [51]). Lastly, *OsTBP2* is linked to *LOC_Os06g48030*, encoding a peroxidase precursor, suggesting that *OsTBP2* may also regulate the detoxification enzyme to scavenge reactive oxygen species (ROS) occurring under salt stress [52,53].

*LOC_Os07g42400* (*OsGATA23*) is a gene in the GATA transcription factors family. We found that *OsGATA23* has significant changes in the expression profile between genes and five other genes in the networks; two genes have a positive different correlation, and three genes have a negative different correlation (Figure 7b). *OsGATA* proteins were found to emerge as transcription factors in response to multi-stress, including salinity stress [30]. Particularly, *OsGATA8*, localized in *Saltol* QTL, which is the major QTL contributing to salt tolerance in rice, has been shown to play roles in ion and ROS homeostasis, including yield maintenance under salt stress conditions [54]. *OsGATA16* was found to be a positive regulator for cold tolerance in rice by repressing some cold-related genes. Moreover, it was shown to be induced by ABA [55]. Gupta and colleagues [30] reported a contrast in the transcript abundance level of individual GATA members in rice genotypes, IR64 (salt-sensitive) and ‘Pokkali’ (salt-tolerant), in response to distinct abiotic stresses such as salinity, drought, and exogenous ABA. These findings support the possibility of *OsGATA23* role in salt tolerance for further investigation. All the genes connected to *OsGATA23* were shown to connect with *OsTBP2* (Figure 7a,b). These genes are *OsGAP* (*LOC_Os07g31720*), *LOC_Os12g14220*, *LOC_Os06g48030*, *LOC_Os03g55389* and *LOC_Os09g35960*. These suggest that both *OsTBP2* and *OsGATA23* respond to salt stress in the same pathway.

*LOC_Os01g13030* (*OsIAA3*) is an auxin-induced gene in the auxin-responsive Aux/IAA gene family. *OsIAA3* has significant changes in the expression profile and connects to six other genes in the networks; only one gene has a positive different correlation, and five genes have a negative different correlation (Figure 7c). Auxin-induced genes are well known as one of the gene families that play a critical role in response to auxin in rice [56,57,58]. Moreover, the auxin response family members were found to be regulated by other transcription factors, such as the MYB transcription factor, the bHLH transcription factor, and the heat shock transcription factor (HSF) [59,60,61]. *OsIAA3* is located in MQTL1.1, which refers to the traits of spikelet fertility, grain number per panicle and 1000-grain weight [62]. It was reported to regulate root growth and grain size in rice [63,64,65,66]. The prediction of *OsIAA3* as one of the key genes here suggests its role in rice yield maintenance under salt stress conditions. *OsIAA3* was predicted to connect with six genes, most of which were consistent with the connected genes with *OsTBP2* and *OsGATA23*. These are *LOC_Os03g55389*, *LOC_Os06g48030*, *LOC_Os07g31720*, *LOC_Os08g12850* and *LOC_Os12g14220*, suggesting that *OsIAA3* functions in the same pathway as *OsTBP2* and *OsGATA23*. *LOC_Os01g12320*, encoding *OsGELP12* or GDSL esterase/lipase protein 12 is the only gene connected to *OsIAA3*, but neither to *OsTBP2* nor *OsGATA23*. GELPs in *Arabidopsis* were reported to be involved stomatal development, early wax biosynthesis and stomatal cuticular ledge formation [67]. In rice, suppression of *OsVPE3* decreased stomatal length and increased salt tolerance [68]. This information suggests the possibility of the involvement of *OsIAA3* in stomatal response through the function of the *OsGELP12* gene.

*LOC_Os05g34050* (*OsbZIP39*) is a bZIP transcription factor. *OsbZIP39* has negative significant changes in the expression profile compared to three other genes in the networks (Figure 7d). *OsbZIP39* has been identified as an ER stress sensor transducer. It is predicted to contain a transmembrane domain and is localized in the ER. The truncated form resulting from proteolytic cleavage can translocate to the nucleus, leading to the regulation of other genes [69]. Members of the bZIP transcription factor family are involved in many biological processes and play essential roles in abiotic stresses, including salt dress in plants [70,71,72,73]. Studies have demonstrated that bZIP transcription factors are extensively involved in rice’s abiotic stress response and ABA signaling, primarily in various abiotic stresses [74,75,76,77,78]. Only three genes were predicted to interact with *OsbZIP39*, namely *LOC_Os06g48030*, *LOC_Os07g31720*, and *LOC_Os12g14220*. These genes were also found to be connected with *OsTBP2*, *OsGATA23*, and *OsIAA3*, suggesting the involvement of these transcription factors in the same pathway of salt stress responses. The function of *OsbZIP39* as an ER stress sensor supports its interaction with *OsTBP2* and *OsIAA3*, as these two genes were predicted to interact with *LOC_Os08g12850*, encoding reticulon-like protein (Figure 7a,c).

*LOC_Os09g29930* (*OsBIM1*) is one of the bHLH transcription factors. *OsBIM1* exhibits negative changes in expression profiles compared to four other genes in the networks (Figure 7e). It has been reported as a positive regulator in brassinosteroid (BR) signaling, responding to rice’s BR treatment, which increases leaf inclination [79]. The changes in leaf angle are proposed to be a part of a stress avoidance mechanism [80]. Using a digital sensor to measure real-time leaf movement, it was found that the leaf angle of salt-stressed tomato plants was higher, and circadian leaf oscillations faded out faster than controls, suggesting adaptation of leaf angle during salt stress [81]. Moreover, many bHLH transcription factors in plants have been reported to be involved in stress responses, such as drought, salt, and cold stress [82,83]. The discovery of this gene’s involvement in the salt stress response suggests that *OsBIM1* may play a role in morphological adaptation to salt stress. Interestingly, in addition to its connection with *LOC_Os06g48030*, *LOC_Os07g31720*, and *LOC_Os12g14220*, which suggests a response in the same pathway as other previously mentioned transcription factors, *OsBIM1* was predicted to connect with *LOC_Os01g28989*, encoding BR-signaling kinase 1 (BSK1). This supports the action of brassinosteroidsin salt stress response.

*LOC_Os10g10990* encodes a transcription initiation factor IIF. We found this gene exhibits significant negative changes in expression profiles compared to 10 other genes in the networks (Figure 7f). *LOC_Os10g10990* has been reported as the target gene for downregulation by miRNA under salt stress conditions [84]. It has also been shown to undergo alternative splicing under heat stress in rice [85]. Further investigation of this gene under salt stress response can be performed to support its role in salt tolerance in rice. This gene is connected with genes that are connected to the previous mentioned transcription factors, namely *LOC_Os06g48030*, encoding a peroxidase precursor, *LOC_Os07g31720* (*OsGAP*), *LOC_Os08g12850*, *LOC_Os12g14220* and *LOC_Os01g28989* (*OsBSK1*), suggesting that *LOC_Os10g10990* plays a role in the same pathway as other TFs in this report. Moreover, the gene strongly linked to this transcription factor that does not connect to other TFs in this report is *LOC_Os08g01670*, encoding pectin methylesterase inhibitor 28 (*OsPMEI28*). In *Arabidopsis*, *PMEI31* positively modulates salt tolerance [86]. *Arabidopsis* mutants with reduced expression of a *PMEI* gene (*At1g62760*) show reduced sensitivity to salt stress [87], while overexpression of *AtPMEI13* could increase salt tolerance. The overexpression of *PMEI1* from the cryophyte, *Chorispora bungeana* led to a decrease in freezing tolerance but an increase in salt tolerance [88]. This suggests that this action of *LOC_Os10g10990* responds to salt stress, not only within the cell, but also at the plant cell wall.

### 2.7. Salt-Stress Response Mechanisms

Based on the information regarding the predicted gene modules with 6 central transcription factors, *OsTBP2*, *OsGATA23*, *OsIAA3*, *OsbZIP39*, *OsBIM1*, and *LOC_Os10g10990*, it has been demonstrated that all these TFs regulate salt stress responses by sharing the same signal transduction pathway. All TFs interact with *OsGAP*, which previously reported to receive the salt stress signal from *IP3* [45]. *CK2* also plays a role in the signaling cascade and interacts with *OsTBP2*, *OsGATA23*, and *OsIAA3*, while *OsRab/GAP* interacts only with *OsTBP2* and *OsGATA23*.

**Figure 8:**
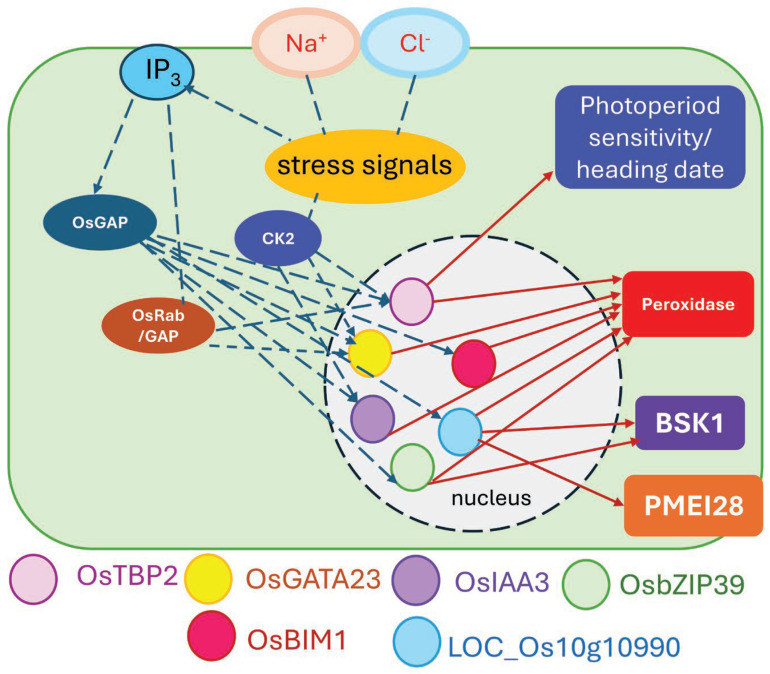
Model of salt stress response leading to salt tolerance in rice based on the predicted transcription factors, *OsTBP2*, *OsGATA23*, *OsIAA3*, *OsbZIP39*, *OsBIM1*, and *LOC_Os10g10990*.

All TFs show a connection with peroxidase precursor protein, suggesting that adaptation to oxidative stress is part of salt tolerance mediated by these TFs. It is worth mentioning that *OsTBP2* was previously reported to be involved in heading date and photoperiod sensitivity, which could be part of the adaptation to salt stress. Two of the identified TFs, *OsbZIP39* and *LOC_Os10g10990*, interact with *BSK1*, suggesting the role of bassinosteroids in the salt tolerance. Lastly, *LOC_Os10g10990* was shown to connect with *PMEI28*, which was predicted to function at the cell wall. However, no experimental evidence has been directly shown for the function of *OsPMEI28*. The model of the interaction and regulation of the predicted genes is shown in Figure 8.

## 3. Discussion

Genomic approaches have proven successful in identifying markers that respond to salinity in different rice varieties under varying salt concentrations. Recently, transcriptomics has emerged as a widely-used method for investigating comprehensive changes in the transcriptome across various biological conditions. This method enables us to understand how genes are expressed under normal and salt stress conditions. Both microarrays and high-throughput RNA sequencing have been extensively utilized to study the biological role of genes under salinity, comparing their expression profiles with those under normal conditions in rice through differential transcriptome analysis. Several studies have identified candidate genes associated with salt tolerance and have provided insights into the mechanisms underlying rice responses to salt stress. However, only a few of these genes have been successfully employed in breeding programs to develop salt-tolerant rice varieties [6,7,89]

This study integrated gene expression data from previous microarray and RNA sequencing experiments involving salt stress across multiple rice cultivars sourced from the GEO database [90]. The gene expression data for salt-sensitive and salt-tolerant rice varieties were separated into two groups and subjected to network analysis to identify potential critical genes in the contrasting rice varieties under normal and salt-stress conditions. By combining gene expression data, we investigated genes that exhibit significance in salt-stress responses, specifically within salt-tolerant rice varieties when compared to salt-sensitive rice varieties. Additionally, this approach enables us to capture transcription factors that contribute to specific salt tolerance mechanisms and may influence other genes in the system.

Our results indicate that both salt-sensitive and salt-tolerant rice cultivars process numerous important genes involved in salt-stress responses. Interestingly, we observed a significant overlap of important genes between the two groups. This suggests that rice, regardless of its salt sensitivity or tolerance, inherently possesses mechanisms to respond to salt stress. However, salt-sensitive rice cultivars may lack certain key genes necessary to effectively induce salt stress resistance. Identifying salt-tolerant genes necessitates comparing two contrasting rice cultivars, specifically those with divergent phenotypes, to detect highly potential genes that confer tolerance to salt stress, distinct from those found in salt-sensitive rice varieties.

Much research on mapping quantitative trait loci (QTL) for salt tolerance in rice has detected several molecular marker intervals or loci and identified many candidate genes within those intervals. Therefore, it is necessary to identify the key important genes to narrow down the list. Interestingly, through the list of high-centrality genes from the salt-tolerant networks in our analysis, we discovered that many genes were identified across different chromosomes associated with the QTL mapping study for salt tolerance in rice. Moreover, 86 high-centrality genes are located within the meta-QTL markers.

With the increasing availability of expression data from multiple experiments, we have identified numerous common genes and genes that are specifically important for salt-tolerant rice. This enables us to screen suitable candidate genes for the genetic improvement of susceptible rice cultivars. While many of the important genes identified in this study are novel, it is worth noting that several genes in our analysis belong to QTL loci that have been previously found to respond to stress in various plants, including rice cultivars. These key genes serve as important references for further studies on the functions of salt stress response in rice plants and hold the potential for developing salt-resistant rice cultivars. Furthermore, these results contribute to a deeper understanding of the complex mechanism involved in rice and guides further analysis and breeding efforts to enhance salt tolerance in rice.

## 4. Materials and Methods

### 4.1. Identification of Transcription Factors Involving Various Significantly Different Correlations between the Networks

All gene expression datasets related to salinity tolerance in rice (from 2005 to 2023) were obtained from the NCBI’s Gene Expression Omnibus (GEO database; https://www.ncbi.nlm.nih.gov/geo/) (accessed on 28 August 2023) [90]. We retrieved experiments that performed transcriptome analyses (RNA) of salt-tolerant and salt-sensitive rice cultivars under control and salt-stress conditions in both microarray and RNA sequencing data. We collected eight relevant datasets for microarray experiments: GSE3053, GSE4438, GSE13735, GSE14430, GSE16180, GSE21651, GSE41650, and GSE48395. All experiments except GSE48395 utilized the Affymetrix Rice Genome arrays platform (GPL2025), which consisted of 57,381 probes, to determine the expression profiles of total RNA. GSE48395 utilized the Agilent-042118 MSU7–Rice platform (GPL17380), the Agilent Technologies, Inc., Santa Clara, CA, USA. To align the probe IDs with gene names in the MSU system [91], we used OryzaExpress, a database integrated with omics data from public databases [92]. We calculated the gene average from multiple probes to consolidate gene expression values. In addition, three experiments involving high throughput sequencing for expression profiling were utilized: namely GSE60287, GSE119720, and GSE133480.

From all experiments, we assigned samples of rice cultivars from each experiment into two groups: salt-sensitive and salt-tolerant. We obtained 12 and 15 datasets in the salt-sensitive and salt-tolerant groups, respectively. The salt-sensitive group comprised IR28, IR29, IR64, ‘Nipponbare’, MI48, m103, and a salt-sensitive rice cultivar, while the salt-tolerant group consisted of ‘Pokkali’, ‘Nonabokra’, CSR27, CSR28, FL478, IR63731, ‘agami’, and a salt-tolerant rice cultivar. Next, we combined each dataset for each group and retained only genes found in all datasets. We identified 24,975 genes expressed among all datasets in the salt-sensitive and salt-tolerant groups. We then rescaled the gene expression of each sample to range from 0 to 100 using the equation—$(\left(x-\min\left(x\right)\right)/\left(\max\left(x\right)-\min\left(x\right)\right))\times 100$, where *x* is the expression value of each gene. Subsequently, we filtered genes with an expression value of less than or equal to 1 in at most 50% of all samples for each group to select genes highly expressed in at least 50% of samples, over those low-expressed in some samples. This filtering process is based on both biological and statistical considerations, as genes with low expression across samples are unlikely to be significant. Moreover, genes must be expressed at some minimal level before they are likely to be translated into a protein or considered biologically important [93]. Finally, we obtain 7135 genes and 77 samples of expressed genes in the salt-sensitive group, and 8274 genes and 101 samples of expressed genes in the salt-tolerant group. Further details of the data and sources are provided in Table S1.

### 4.2. Construction of Weighted Gene Co-Expression Network and Identification of High Centrality Genes

Based on gene expression profiles in the salt-sensitive and salt-tolerant groups, we detected outliers using hierarchical clustering. This clustering was carried out to cluster the samples by average, resulting in 72 samples for salt-sensitive group and 96 samples for the salt-tolerant groups. We then constructed a gene co-expression network for each rice group across all conditions using weighted correlation network analysis (WGCNA) [18,20]. We first computed the Pearson correlation of the expression profiles between gene pairs. To preserve the sign of the correlation, we used a signed network type to reflect the sign of the correlation of their expression profiles. The similarity of the signed co-expression network between gene *i* and gene *j* is defined as $s_{ij}^{signed}=\frac{1+cor(i,j)}{2}$, which takes on a value between 0 and 1. Nodes strongly negatively correlated were considered unconnected ($s_{ij}^{signed}$ = 0), while the signed co-expression measure of two genes with zero correlation remains 0.5. Next, the similarity matrix was transformed into an adjacency matrix by raising it to the power of soft thresholding, defined as $a_{ij}={\left(s_{ij\;}^{signed}\right)}^{\beta }$, quantifying the strength of connections between genes, with a higher value of beta (*β*) indicating stronger connections [18,20]. To determine the appropriate *β* parameter, we utilized the function pickSoftThreshold, which aimed to establish a scale-free network topology by maximizing the R-squared (R^2^) value through linear regression analysis while simultaneously minimizing the loss of connections to maintain a high average number of connections. We selected *β* equal to 22, which yielded an R^2^ value greater than 0.5. This selected *β* parameter was then used to calculate the adjacency matrix and construct the weighted co-expression networks for the salt-sensitive and salt-tolerant datasets, respectively. We then computed the topological overlap similarity (TOM) to measure both the strength of direct connections and the number of shared neighbors between gene pairs. The calculation of TOM is defined as

$${TOM}_{ij}^{signed}=\frac{\left|a_{ij}+\sum_{u\neq i,j}a_{iu}a_{uj}\right|}{min\left(k_{i},k_{j}\right)+1-\left|a_{ij}\right|}$$

where $k_{i}$ and $k_{j}$ are the connectivity of node *i* and node *j*, $k_{i}=\sum_{u\neq i}\left|a_{ui}\right|$. If genes have a substantial number of shared neighbors, their TOM value is high [18,20]. We applied a cutoff of 0.4 for the TOM to export each network to Cytoscape version 3.10.1 [21]. Each network was then analyzed using the Analyzed Network tool in Cytoscape to measure degree centrality (DG), betweenness centrality (BW), closeness centrality (CN), and clustering coefficient (CC) of genes in the network. Key genes were determined based on their centrality measures in both networks.

### 4.3. Identification of Genes with High Differential Correlations between Networks

To identify significant differences in correlations between networks, we first merged lists of selected genes from the salt-sensitive and salt-tolerant groups. We then retrieved the expression profile of genes present in both groups for further analysis. A complete weighted network was then constructed using the expression data from the salt-sensitive and salt-tolerant groups. The edges connecting two genes were calculated using Pearson correlation. To examine the differences in correlations between the salt-sensitive and salt-tolerant networks, we utilized the DiffCorr package in R [19]. Significance testing for different correlation coefficients among all pairs of genes in the two networks was performed using Fisher’s z-test. To control false discoveries, the local false-discovery rate (FDR) was employed. A threshold of at least 0.7 for the significantly different correlation between networks and an FDR of 0.05 for the different correlations were used to select the significantly different edges.

### 4.4. Gene Ontology Enrichment Analysis

Genes were subjected to GO enrichment analysis using the Singular Enrichment Analysis (SEA) tool available on AgriGO v2.0 (http://systemsbiology.cau.edu.cn/agriGOv2/) (accessed on 15 December 2023). AgriGO v2.0 is a web-based tool and database specifically designed for plant gene ontology analyses [94]. For this analysis, we used the MSU version 7.0 from the TIGR plant repeat databases as a reference for rice [95]. Fisher’s tests were employed to assess the significance of GO terms, and false discovery rates (FDRs) were calculated for the multiple hypothesis adjustments to effectively control the type I error. A threshold of *p*-value and FDR less than or equal to 0.05 was applied to identify significant GO enrichment.

## 5. Conclusions

In this study, we identified potential candidate genes to enhance susceptible rice cultivars through gene co-expression network analysis. By constructing and comparing two gene co-expression networks, we investigated candidate genes using gene-expression data from multiple salt-sensitive and salt-tolerant rice varieties. Our analysis revealed six transcription factors with high-centrality values in the salt-tolerant networks. These factors actively involved in many changes in the expression pattern between gene pairs, when comparing salt-sensitive and salt-tolerant rice. Moreover, we identified 86 genes located in the meta-QTL intervals from previous research on rice salt tolerance. While many key genes identified in this study are novels, several genes are associated with protein families that respond to stress, consistent with findings in various plant studies, including rice cultivars. These key genes may serve as crucial references for further studies on the functions of salt-stress responses in rice plants, facilitating in the development of salt-resistant rice cultivars. Moreover, these results contribute to our understanding of the complex mechanism in rice and hold potential for breeding salt-tolerant rice.

## Figures and Tables

**Table 1:** Network properties of salt-sensitive network and salt-tolerant network.

Properties	Salt-Sensitive Network	Salt-Tolerant Network
The network diameter	7	4
The network radius	4	2
The average number of neighbors	861.908	833.153
The average shortest path length	1.966	1.902
The network density	0.183	0.162
The network heterogeneity	0.999	1.098
The network clustering coefficient	0.824	0.851

**Table 2:** List of high-degree genes specific only in salt-sensitive or salt-tolerant networks.

Genes	Function	DG in the Salt-Sensitive Network	DG in the Salt-Tolerant Network
*LOC_Os09g32430*	NADH: ubiquinone oxidoreductase	3401	0
*LOC_Os06g05150*	expressed protein	0	4320
*LOC_Os02g42550*	expressed protein	0	4050
*LOC_Os06g42430*	DUF1950 domain containing protein	0	3956
*LOC_Os01g17260*	TGA transcription factor, bZIP transcription factor, de-fense response, multiple abiotic stress tolerance	0	3882
*LOC_Os06g21580*	HMG-I and HMG-Y, DNA-binding domain containing protein	0	3708
*LOC_Os07g42395*	DNA-directed RNA polymerase II subunit RPB9	0	3544
*LOC_Os03g60360*	protein kinase PKN/PRK1, effector	0	3384
*LOC_Os03g10200*	ethylene-responsive element-binding protein	0	3380
*LOC_Os02g45920*	expressed protein	0	3336
*LOC_Os02g57370*	DHHC zinc finger domain containing protein	0	3325
*LOC_Os08g01660*	mago nashi	0	3162
*LOC_Os10g37690*	inner membrane protein	0	2962
*LOC_Os09g28590*	AGAP010609-PA	0	2898
*LOC_Os03g37330*	KIN, antigenic determinant of recA protein	0	2892
*LOC_Os02g48590*	expressed protein	0	2887
*LOC_Os07g27760*	expressed protein	0	2884
*LOC_Os03g26090*	GPI-anchored wall transfer protein 1	0	2824
*LOC_Os03g55784*	tumor necrosis factor superfamily, member 5-induced protein 1	0	2779
*LOC_Os10g32870*	L11 domain containing ribosomal protein	0	2749
*LOC_Os01g46580*	actin-related protein 2/3 complex subunit 2	0	2739
*LOC_Os03g63140*	L11 domain containing ribosomal protein	0	2735
*LOC_Os03g46610*	DEAD-box ATP-dependent RNA helicase	0	2733
*LOC_Os06g05250*	GTP-binding protein GUF1	0	2721
*LOC_Os02g10050*	expressed protein	0	2698
*LOC_Os05g23530*	expressed protein	0	2684

**Table 3:** List of high closeness centrality (CN) genes only in salt-sensitive or salt-tolerant networks.

Genes	Function	CN in the Salt-Sensitive Network	CN in the Salt-Tolerant Network
*LOC_Os04g52450*	γ-Aminobutyric acid transaminase	1	0
*LOC_Os03g23980*	NAD dependent epimerase/dehydratase family protein	0.8257	0
*LOC_Os09g32430*	NADH: ubiquinone oxidoreductase	0.7693	0
*LOC_Os06g05150*	expressed protein	0	0.8577
*LOC_Os02g42550*	expressed protein	0	0.8181
*LOC_Os06g42430*	DUF1950 domain containing protein	0	0.8087
*LOC_Os01g17260*	TGA transcription factor, bZIP transcription factor, defense response, multiple abiotic stress tolerance	0	0.7966
*LOC_Os06g21580*	HMG-I and HMG-Y, DNA-binding domain containing protein	0	0.7783
*LOC_Os07g42395*	DNA-directed RNA polymerase II subunit RPB9	0	0.7570
*LOC_Os10g42500*	PAP fibrillin family domain containing protein	0	0.7565

## Data Availability

All gene expression datasets in this study were obtained from the NCBI’s Gene Expression Omnibus (GEO database; https://www.ncbi.nlm.nih.gov/geo/) (accessed on 17 February 2024).

## Supplementary Materials

The following supporting information can be downloaded at: https://www.mdpi.com/article/10.3390/genes15030316/s1, Table S1: The lists of high-degree genes, degree centrality, and description of each gene in the salt-sensitive (Table S1.1) and salt-tolerant network (Table S1.2); Table S2: The significant GO terms of high-degree genes in the salt-sensitive network (Table S2.1) and salt-tolerant network (Table S2.2); Table S3: The lists of high-closeness genes, closeness centrality, and description of each gene in the salt-sensitive (Table S3.1) and salt-tolerant network (Table S3.2); Table S4: The significant GO terms of high-closeness genes in the salt-sensitive network (Table S4.1) and salt-tolerant network (Table S4.2); Table S5: List of high-centrality genes that are associated with the meta-QTLs study of salt tolerance in rice; Table S6: The list of genes involved in transcription activities, their significantly different correlations between the two networks, and the description of each gene in the salt-sensitive and salt-tolerant network.
